# New Formulations of Natural Substances Against Insect Pests

**DOI:** 10.3390/insects16070646

**Published:** 2025-06-20

**Authors:** Priscilla Farina, Barbara Conti

**Affiliations:** 1 Department of Agriculture, Food and Environment, University of Pisa, Via del Borghetto 80, 56124 Pisa, Italy; 2 Nutrafood, Research Center Nutraceuticals and Food for Health, University of Pisa, Via del Borghetto 80, 56124 Pisa, Italy

Arthropod pest populations can significantly impact a variety of anthropogenic activities, including food production and human and animal health. Managing pest populations has been a cornerstone of all civilisations since the dawn of time. Although only a small percentage of insects and mites—1–3% of species—are considered harmful, they need to be managed swifty and precisely by integrating all available low environmental impact tools (e.g., monitoring, prevention, biological, physical, mechanical, agronomic, and chemical means), not forgetting the biorational pesticides. Biorational options, often derived from natural substances of plant, microbial, or mineral origin, are specifically designed to minimise harm to the environment and non-target organisms while effectively controlling pest infestations when strictly necessary.

Building on the experience acquired with the previous Special Issue “Natural Substances against Insect Pests: Assets and Liabilities”, we decided to move forward to “New Formulations of Natural Substances against Insect Pests”. This subsequent Special Issue focuses on the isolation, chemical and biochemical characterisation, and formulation of natural substances, as well as their use as toxicants, growth and/or reproduction inhibitors, repellents, and deterrents towards arthropod pests. It also explores their mode(s) of action and the associated risks and benefits of their use. In total, we have gathered seven original research articles and one review paper.

Arthropod pests of medical, veterinary, and agricultural interest encompass the blood-feeding mosquitoes and ticks, both active vectors of infectious diseases, as well as phytophagous insect and mite species that feed on various plant tissues, from the field to storage facilities. The target pests addressed in this Special Issue include (i) mosquitoes *Aedes albopictus* [Contribution 1] and *Anopheles arabiensis* (Diptera Culicidae); (ii) the tsetse fly *Glossina fuscipes* (Diptera Glossinidae) [Contribution 2]; (iii) hard ticks *Dermacentor reticulatus*, *Ixodes ricinus* [Contribution 3], and *Rhipicephalus* spp. (Acari Ixodidae) [Contribution 2]; (iv) the green peach aphid *Myzus persicae* (Hemiptera Aphididae) [Contribution 4]; (v) the pineapple mealybug *Dysmicoccus brevipes* (Hemiptera Pseudococcidae) [Contribution 5]; (vi) the black cutworm *Agrotis ipsilon* (Lepidoptera Noctuidae) [Contribution 6]; (vii) the cowpea weevil *Callosobruchus maculatus* (Coleoptera Bruchinae) [Contribution 7].

Among the botanicals featured, essential oils distilled from plants from all over the world are the most represented group in this Special Issue. This marked interest reflects the intense research focus on essential oils over the past 40 years. Examples include (i) the essential oil of *Carum carvi* (Apiaceae) seeds from Latvia, tested as an aphid repellent [Contribution 4]; (ii) the essential oils of Ecuadorian *Aloysia citrodora* (Verbenaceae) leaves and *Bursera graveolens* (Burseraceae) stems, showing repellent, deterrent, and larvicidal activities against mosquitoes [Contribution 1]; (iii) *Origanum majorana* and *Salvia rosmarinus* (Lamiaceae) essential oils from leaves of plants grown in Egypt, shown to affect the enzymes ATPases, α-esterase, and glutathione S-transferase in moth larvae [Contribution 6]; (iv) a selection of 11 essential oils from Poland mixed in five different combinations and demonstrating anti-tick properties [Contribution 3].

Plant extracts prepared with solvents of varying polarities are another group of botanicals commonly tested. Ethanol extracts of leaves and fruit from *Nicotiana tabacum* and *Nicotiana rustica* (Solanaceae), *Azadirachta indica* and *Melia azedarach* (Meliaceae), and *Thuja orientalis* (Cupressaceae) from Pakistan were herein tested as toxicants, deterrents, and repellents for managing bruchid beetle infestation of pulses [Contribution 7].

Ethyl formate, a volatile organic compound naturally released by many fruits, was proposed and tested as a substitute for methyl bromide in the fumigation of pineapples against mealybugs [Contribution 5]. As a further biorational option, the fungal isolates ICIPE 7 and ICIPE 30 of *Metarhizium anisopliae* proved effective for the integrated and combined management of ticks (both nymphs and adults), disease-transmitting mosquitoes, and tsetse flies infesting cattle [Contribution 2].

Formulation attempts included the use of single and multiple surfactants with various hydrophilic-lipophilic balance values, applied either alone [Contribution 4] or combined with biopolymers like sodium carboxymethyl cellulose and chitosan to create oil-in-water emulsions of essential oils [Contribution 3]. Chitosan, a polysaccharide derived from the deacetylation of chitin, is the subject of the included review, which highlights its role as a valuable tool in pest control. As reported, in addition to solutions and nanoparticles loaded with essential oils, chitosan has been successfully applied onto leaves, fruits, wood, cardboard, and paper, either simply solubilised, as a film form, in metal complexes, or even formulated with nematodes, providing promising control of agricultural, veterinary, and public health pests [Contribution 8].

In conclusion, this Special Issue offers just a taste of the vast world that has emerged in recent years around biorational solutions for arthropod pest control. Further investigations are warranted to optimise the formulations of natural substances and select concentrations that show concrete effectiveness under field and operative conditions, before they can be widely adopted in practice. While additional efforts are required from the scientific and technological communities, the basic research and starting points for applied approaches are fortunately not lacking.

## Data Availability

No datasets were generated or analysed.

